# Influence of Different Regimes of Moderate Maternal Feed Restriction during Pregnancy of Primiparous Rabbit Does on Long-Term Metabolic Energy Homeostasis, Productive Performance and Welfare

**DOI:** 10.3390/ani11092736

**Published:** 2021-09-19

**Authors:** Carlota Fernández-Pacheco, Pilar Millán, María Rodríguez, Nora Formoso-Rafferty, Ana Sánchez-Rodríguez, Pedro L. Lorenzo, María Arias-Álvarez, Rosa M. García-García, Pilar G. Rebollar

**Affiliations:** 1 Department of Physiology, Faculty of Veterinary, Complutense University of Madrid, Avenida Puerta de Hierro s/n, 28040 Madrid, Spain; cafpmartorell@ucm.es (C.F.-P.); pmillanp@vet.ucm.es (P.M.); anasanro.vet@gmail.com (A.S.-R.); plorenzo@vet.ucm.es (P.L.L.); rosa.garcia@vet.ucm.es (R.M.G.-G.); 2 Department of Agrarian Production, ETSIAAB, Technical University of Madrid, Ciudad Universitaria s/n, 28040 Madrid, Spain; maria.rodriguez@pigchamp-pro.com (M.R.); nora.formosorafferty@upm.es (N.F.-R.); 3 Department of Animal Production, Faculty of Veterinary, Complutense University of Madrid, Avenida Puerta de Hierro s/n, 28040 Madrid, Spain; m.arias@vet.ucm.es

**Keywords:** rabbit, feed intake, free tri-iodothyronine, thyroxine, insulin, glucose, corticosterone, NEFA, fetus, placenta

## Abstract

**Simple Summary:**

In rabbit farms, the main production costs come directly from food supplies. Although the reproductive outcomes in this species are acceptable, the results are worse when it comes to primiparous rabbits, so it is recommended that insemination be carried out post-weaning. By avoiding the overlap of the second gestation and the first lactation, better fertility results are expected. Still, despite this, the rabbits garner more adipose tissue than desired, and the productive efficiency deteriorates in the long term. The purpose of this study was to evaluate the influence of different periods of moderate feed restriction (one, two or three weeks) applied during the second pregnancy of primiparous does. We studied fetoplacental development, productive parameters, metabolism and possible stress indicators. Results showed that the voluntary feed intake of dams increased right after feed restriction. No permanent alterations were found in reproductive outcome, metabolism or welfare of does, meaning that this feeding strategy could be successfully applied in rabbit farms.

**Abstract:**

In this study, a maternal feed restriction (MFR; 105 g/d) in primiparous rabbit does was applied from day 0 to 7 post artificial insemination (AI) (R07, n = 96), from day 7 to 21 post AI (R721, n = 92), from day 0 to 21 post AI (R021, n = 94) or fed ad libitum during whole pregnancy (Control, n= 92). Feed intake (FI) was measured after MFR was over. On day 28 of gestation, fetoplacental development was evaluated (n = 11/group) and the productive parameters of the remaining dams were analyzed. Plasma free tri-iodothyronine (T3) and thyroxine, glucose, insulin, non-esterified fatty acids (NEFA), and corticosterone were analyzed during gestation and lactation (n = 5/group). After MFR, all groups significantly increased their voluntary FI. The longer MFR was, the lower the weight and length of the fetuses, but no long-term effects over litter performance were observed. R021 groups had the lowest T3 and the highest NEFA concentrations during pregnancy and showed insulin resistance at the end of gestation, but during lactation, energy homeostasis was balanced in all groups. MFR did not affect corticosterone concentrations. In conclusion, the ration setting applied slightly involved the energy homeostasis and metabolism of the animals, but their overall metabolic condition, productive performance and welfare were not compromised.

## 1. Introduction

In rabbit farms, dam feeding represents roughly a third of the total feed costs (3.7% and 31.7% for replacement and reproductive does, respectively) [1]. Moreover, the number of kits born alive and the feed conversion rate during fattening are of high economic importance. These traits depend directly on maternal nutrition during pregnancy and lactation. Artificial insemination (AI) of does is usually applied on Day 4 or 11 post-partum, while females are lactating [2]. Alternative breeding strategies can be applied to alleviate the negative energy balance that befalls after first parturition with concurrent gestation and lactation of primiparous does, which are still completing their body growth [3]. One of them is delaying the interval parturition-AI until weaning (Day 30 post-partum), applying a reproductive extensive rhythm [4]. This improves reproductive outcome [5], but considering the first weeks of pregnancy do not involve a very high energy expenditure and it is considered an anabolic period [6,7], the important risk of fattening exists due to ad libitum feeding of post-weaning inseminated does. Nonetheless, it is still a critical phase that can influence the rate of embryonic implantation and the development of the placenta, which are limiting factors for good fetal growth. Moreover, the mentioned fattening risk increases if these dams do not become pregnant (pregnancy diagnosis is usually performed on Day 10–14 post AI). Thus, feeding strategies as a subject of study in these animals include applying different levels of maternal feed restriction (MFR) during diverse periods of pregnancy.

MFR has had diverse targets to avoid fattening and high mortality around parturition [8], to determine the competition for materno-fetal resource partitioning [9], to increase voluntary feed intake at the beginning of lactation, or to allow a longer productive life of rabbit does [10]. López-Tello et al. [9,11] verified that undernourishment in dams (50% MFR) during the entire pregnancy or during preimplantation period (1st week) has adverse effects on the fetoplacental unit (reduced fetal crown-rump lengths, asymmetrical growth and high apoptotic rates at the decidua and labyrinth zone). In this sense, it has also been described that if the daily amount of food provided increases to 60% of the dam’s total voluntary intake, negative effects are observed only in the second half of gestation. In contrast, if MFR is applied in the first 15 days of pregnancy, no adverse effects are observed on the viability or weight of the kits at birth [12]. Moreover, ad libitum refeeding in the last third of gestation has been shown to improve the energetic status of the mothers before parturition [13], precisely when fetal needs are most significant. It also reduces the negative effects of subsequent lactation on their reproductive function (poor ovulatory response and increased delivery-fertile insemination interval) and improves the weight and viability of the kits.

What we can introduce about the application of MFR is largely based upon our previous experimental studies [14,15], in which we have described the metabolic consequences observed in mothers and offspring subjected to a 60% MFR during three weeks of pregnancy (from day 0 to 21). This MFR led to a compensatory feed intake during the last week of pregnancy that offset the effects in the live body weight (LBW) and body reserves of the dams at parturition and the fetal body weight and phenotype at day 28 of pregnancy. These studies on MFR applied during three weeks of pregnancy have been limited to specific comparisons of the metabolic profiles of mothers and fetuses at a particular moment of gestation (day 28).

Thus, in order to gain further understanding of the metabolic changes and energetic modulations of primiparous does subjected to MFR, the novelty of this study has been to apply shorter and more specific periods of restriction during gestation studying the long-term consequences on additional hormones and metabolites. Among others, thyroid hormones are key to the regulation of metabolism and adaptation to fasting. They contribute to both mandatory and adaptive thermogenesis, regulating appetite and energy expenditure [16]. On the other hand, glucose is central to energy consumption. Carbohydrates, lipids, and proteins all ultimately break down into glucose, which serves as the main metabolic fuel of mammals’ cells and the prevalent fuel of the fetus [17]. Likewise, insulin is a primary anabolic hormone secreted in response to increased blood glucose and amino acids following feeding. The major action of insulin is to stimulate and control glucose consumption and exertion. Like other hormones, insulin performs its activities by binding to specific receptors present on many cells distributed all through the body [18]. Additionally, non-esterified fatty acids (NEFA) concentrations reflect the mobilization of body reserves since they are generated from lipolysis in fatty tissue and are an indicators of negative energy balance [19]. Briefly, the main interest of this study was to measure these metabolic pathways and hormones since they are known to have different behaviors when adapting to the specific needs of gestation, fetal development and milk production [20]. Furthermore, and considering animal welfare standards, food or water deprivation is a procedure that can cause pain or distress [21], making the analysis of stress-related hormones mandatory when a feed restriction is applied. It should be noted that in rabbits and rodents, unlike other mammals, the main glucocorticoid analyzed is usually corticosterone rather than cortisol [22].

Therefore, the main goal of this study was to determine in primiparous lactating does the comparative effect of MFR applied during one, two or three weeks of pregnancy followed by ad libitum refeeding on the feed intake of the dams, feto-placental development on day 28 of pregnancy, productive performance, the weekly plasma concentrations of thyroid hormones and corticosterone, and the glucose and lipid metabolism during pregnancy and consequent lactation.

## 2. Materials and Methods

### 2.1. Experimental Design

**Figure 1:**
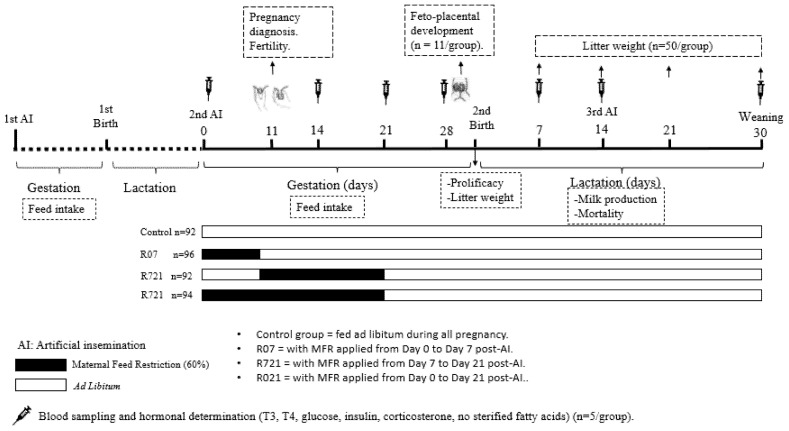
Experimental design.

The animals were housed at the animal facilities of the Technical University of Madrid (Spain), which meet the local, national and European requirements for Scientific Procedure Establishments (RD 53/2013; PROEX 302/15). The experimental design is displayed in Figure 1. New Zealand × California rabbits (18 weeks old), were fed ad libitum during their first pregnancy (length 31 days) and lactation (length 30 days) with a diet containing 16% crude protein, 37% crude fiber, 3.7% fat and 2400 kcal/kg of digestible energy (NANTA, Madrid, Spain). During the first pregnancy, the feed intake of each doe was recorded daily and established in 175 g per animal and day.

After parturition and weaning (day 30 post-partum) these does started a second cycle and were artificially inseminated (AI) with fresh diluted semen (commercial extender, MA 24, Ovejero, León, Spain). Each dose contained at least 20 million spermatozoa in 0.5 mL of semen diluent. Ovulation was induced with gonadoreline at the time of AI (20 μg/doe, i.m.; Inducel-GnRH, Ovejero, León, Spain). At this time (day 0), a total of 374 does were randomly allocated into four groups:

- Control group (n = 92) always fed ad libitum.
- R07 (n = 96) with MFR applied from day 0 to day 7 post-AI.
- R721 (n = 92) with MFR applied from day 7 to day 21 post-AI.
- R021 (n = 94) with MFR applied from day 0 to day 21 post-AI.

MFR was set at 60% of the measured voluntary feed intake of the first pregnancy (105 g/day), in order to maintain their basal metabolic needs. In the fourth week of pregnancy, all animals were fed ad libitum until the end of the study. Pregnancy was diagnosed at day 11 post-AI by means of abdominal palpation and fertility ([no. of does pregnant at day 11 after AI/no. of does inseminated] × 100) was calculated. Feed intake was weekly calculated exclusively in the pregnant does from day 0 until the day of parturition.

### 2.2. Fetoplacental Study

On day 28 of gestation, 44 pregnant does (n = 11 from each group) were weighed and euthanized with an overdose barbiturate (Dolethal, Lab. Vetoquinol, Madrid, Spain) to study fetoplacental development. A ventral laparotomy was performed to count the corpora lutea present in the ovaries and calculate the ovulation rate per animal. Both uterine horns were opened to extract the fetuses and placentas, calculating the rate of viable structures [(number of viable fetuses/number total of fetuses) × 100]. In morphologically viable fetuses, crown–rump length (CRL), biparietal (BPD), occipito-nasal (OND), and thoracic (TD) diameters were measured. Whole fetuses were weighed, and after decapitation, heads and trunks were weighed separately. Fetal organs’ (brain, liver, heart, lungs, digestive tract and kidneys) weights and their ratios (organ weight/fetus weight) were calculated to assess fetal growth patterns. Placentas were weighed both as a whole and separately for the maternal (decidua) from the fetal (labyrinth) part. The length, width and thickness of each of the pieces were recorded. Placental efficiency was calculated by dividing the weight of the fetus between the weight of the whole placenta.

### 2.3. Productive Outcome

The gestation of the remaining rabbit does was carried out, and prolificacy (no. of kits born alive and stillborn per doe) and litter weights at birth were recorded. Litter size was subsequently standardized to 8–12 kits by removing or adding kits within each experimental group.

During lactation, a total number of 50 litters from each experimental group were weighed at day 7, 14 and 21 post-partum. Milk production was estimated using the regression equation developed by Helfenstein et al. [23] as follows: milk production (kg) = 0.75 ± 1.75 LW21 (kg) where LW21 corresponds to litter weight at 21 days of lactation. The day of weaning (day 30 post-partum) the number of weaned kits per doe, litter weight and mortality during lactation period were assessed. At the end of the study, the percentage of culled does (abortions or sudden death) in each experimental group was calculated.

### 2.4. Long-Term Maternal Metabolic and Hormonal Study

In addition, the evaluation of metabolic and hormonal parameters was carried out on blood samples obtained from the marginal ear vein of randomly chosen females. An initial blood sample was taken at the moment of AI (day 0 of pregnancy) from 10 dams before being allocated into the experimental groups. After pregnancy diagnosis, five pregnant animals per group were sampled at three different moments of gestation: day 14 (n = 20), day 21 (n = 20) and day 28 (n = 20) of gestation. Following parturition, samples were taken from lactating females (five per group) at three moments of lactation: day 7 (n = 20) and day 14 (n = 20). On day 14 post-partum, all does were re-inseminated as previously described. Then, on day 30 post-partum, weaning was performed and blood samples were taken from pregnant (n = 20) and non-pregnant (n = 20) does from the third AI. Blood samples were placed in tubes with EDTA as an anticoagulant and centrifuged for 15 min at 1200 g to obtain plasma.

Moreover, the evaluation of energetic homeostasis was performed by measuring free triiodothyronine (T3) and thyroxine (T4) in plasma samples using commercial immunoassays (Demeditec Diagnostics GmbH, Kiel, Germany). The assay sensibility was 0.05 pg/mL for both T3 and T4, and the inter-assay and intra-assay variation coefficients were 9.8% and 3.6% for T3 and 4.9% and 3.3% for T4. Glycemic metabolism was assessed by the determination of insulin and glucose levels. Insulin was determined using a commercial immunoassay (Mercodia Ultrasensitive Insulin ELISA, Mercodia AB, Uppsala, Sweden) with an assay sensitivity of 0.071 mU/L and inter-assay and intra-assay variation coefficients of 6.2% and 4.2%, respectively. Insulin sensitivity was calculated by glucose-to-insulin ratio and the homeostasis model assessment for insulin resistance (HOMA-IR) using the following equation: [insulin (mU/L) × (glucose (mg/dL)/18)]/22.5 [23]. Low HOMA-IR values (<1.96) indicate high insulin sensitivity, whereas high HOMA-IR values (>3) indicate low insulin sensitivity (insulin resistance). Non-esterified fatty acids (NEFA) were selected as the main indicator of lipid metabolism and were measured using a clinical biochemistry assay (Biolabo SAS, Maizy, France), with detection in the range of 0.01–3.0 mmol/L and assay sensitivity 0.050 mmol/L. Corticosterone was used as a stress indicator [22] and was determined using a commercial immunoassay (Demeditec Diagnostics GmbH, Kiel, Germany). The assay sensitivity was 1.63 nmol/L and the inter-assay intra-assay variation coefficients were 6.5% and 2.8%.

### 2.5. Statistical Analysis

SAS software (Statistical Analysis System Institute Inc.; Cary, NC, USA, 2001) was used for the statistical analysis of the data. In all analyses, the doe was considered the experimental unit. Differences between groups in the total feed intake were analyzed by a one-way ANOVA (Proc GLM) with the feed regime as the main effect. The effect of MFR on weekly feed intake during gestation was analyzed by repeated measure analysis (MIXED), with feed regime (Control, R07, R721 and R021), time (weeks 1, 2, 3 and 4), and their interaction as main effects. Ovulation rate, live body weight of does, number, weights and measurements of fetuses and placentas, and placental efficiency on day 28 of pregnancy were analyzed (Proc GLM) with feed regime as main effect and considering the litter size as a covariate. With the same procedure, prolificacy and milk production were analyzed with feed regime as the main effect. A chi squared test (Proc CATMOD) was used to evaluate the effect of MFR on fertility and percentage of culled does. Litter weights during the lactation period were analyzed by repeated measure analysis (Proc MIXED), considering the MFR, the time (birth, 7, 14, 21, and 30 days post-partum) and their interaction in the model. For hormonal and metabolic parameters, due to the blood sampling being carried out in independent randomly chosen rabbit does, a GLM procedure was performed, considering as main effects the treatment (MFR), the time (pregnancy: day 14, 21 and 28 of gestation; and lactation day 7, 14 and 30 post-partum), and the interaction between both. If significant main effects were detected, a Tukey test (for parametric variables) or Kruskal–Wallis (non-parametric variables) were used to compare means among groups, considering the existence of significant differences for a *p* value of less than 0.05. All data are presented as least squares means.

## 3. Results

### 3.1. Feed Intake during Pregnancy

**Figure 2:**
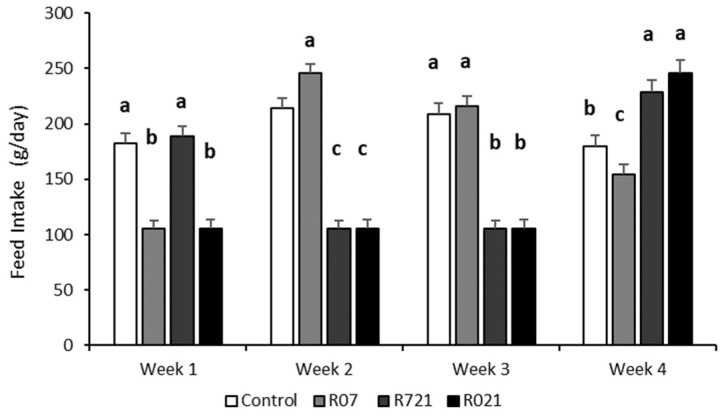
Weekly evolution of the daily feed intake (g/day) in pregnant rabbit does fed ad libitum (Control), restricted to 60% one week from day 0 to 7 (R07), two weeks from day 7 to 21 (R721) and three weeks from day 0 to 21 (R021) of gestation. (a–c): Different letters represent significant differences between experimental groups (*p* < 0.0001).

As expected, whole feed intake during pregnancy was different between groups (*p* < 0.0001). The Control and R07 groups consumed 5854 ± 124.3 g and 5529 ± 109 g, respectively, followed by R721 does (4518 ± 112 g) and the R021 group (4007 ± 106.3 g). The weekly evolution of daily feed intake is shown in Figure 2. Interestingly, the week after MFR, we observed that all groups increased their voluntary feed intake, surpassing the control group, like group R07 during the second week, and groups R721 and R021 during the fourth week of pregnancy.

### 3.2. Fetoplacental Study

Results obtained on day 28 of pregnancy are shown in Table 1. This table is quite revealing in several ways. First, regarding body reserves of does at the end of pregnancy, does restricted for two weeks (R721) presented a lower LBW than Control and R07 groups (*p* = 0.0254), whereas does restricted for three weeks (R021) had an intermediate body weight.

From a reproductive point of view, the number of corpora lutea, total fetuses per doe, implantation rate and fetal viability were high and similar in all groups. In R07 does, there is a tendency (p = 0.0824) to have more fetuses but with lower viability.

Feed restriction significantly affected (*p* < 0.01) the total, head and trunk weights and CRL of fetuses, being lower in those from R721 and R021 does than in the control group, and intermediate in R07 females. Regarding fetal cephalic diameters, BPD was similar in all groups (*p* > 0.05), however OND was higher in Control and R07 does than in R021, being intermediate in R721 (*p* = 0.0032). Fetal thoracic diameter was greater in R07 compared to the other three groups (*p* = 0.0034). Regarding fetal organs, only liver, lung and kidney weights were different between groups (*p* = 0.0097, *p* = 0.0166, *p* = 0.0001, respectively), with those of the Control group being heavier except for the liver.

Weights and dimensions of labyrinth and decidua from euthanized does at day 28 of pregnancy are shown in Table 2. Regarding these data, it is interesting to indicate that the whole placenta of R07 does weighed more than those from the R721 and R021, and the Control group had an intermediate value (*p* < 0.0001). Furthermore, the labyrinth of R07 does tended to be the heaviest (*p* = 0.0809), whilst that of R021 does was the shortest (*p* < 0.0001) and that from R721 the thinnest (*p* < 0.0053). In does subjected to MFR for two weeks (R721), decidua had lower weight (*p* < 0.0001) than the other three groups. The longest decidua was observed in Control does (*p* < 0.0001) and the thinnest was observed in R721 (*p* = 0.0069). Finally, the lowest placental efficiency was observed in R07 does, the highest in R721, and intermediate in Control and R021 does (*p* = 0.0162).

### 3.3. Productive Outcome

Productive outcome is shown in Table 3.

Fertility, kits born alive and stillborn, and litter weight at birth and during lactation were similar between groups (*p* > 0.05). However, after litter adjustment, the effect of MFR on the number of weaned kits and mortality of kits during lactation was significantly different between groups (*p* = 0.0004 and *p* = 0.0325, respectively). Control and R07 does weaned more kits than R721 does, whereas R021 does had an intermediate result. The highest and lowest mortalities during lactation were observed in R021 and R07 litters, respectively.

At the end of the study, the percentage of culled does was 7.6, 4.2, 5.4 and 6.4% in Control, R07, R721 and R021 groups, respectively (*p* > 0.05).

### 3.4. Long-Term Maternal Metabolic and Hormonal Study

Endocrine and metabolic parameters obtained in the experimental groups during pregnancy are shown in Table 4.

Regarding thyroid hormones, the animals subjected to the most extended restriction treatment (R021) had lower plasma T3 concentrations than Control and R07 does (*p* = 0.0196) but similar to R721 ones. Plasma T3 concentrations decreased significantly on days 14 and 21 of pregnancy in relation to day 0 and returned to intermediate values on day 28 (*p* = 0.0026). Plasma T4 concentrations on Days 14 and 21 of pregnancy were also significantly lower than on day 28 (*p* = 0.0001) but similar to that on day 0. MFR did not affect T4 concentrations, T3 to T4 ratio, plasma glucose and HOMA-IR (*p* > 0.05).

However, insulinemia was lower, and glucose-to-insulin ratio was higher in R721 does than in Control and R07 does, but similar to R021 ones (*p* = 0.0225 and *p* = 0.0482, respectively). In addition, plasmatic glucose increased dramatically at the end of pregnancy in all groups (*p* < 0.0001).

The highest and lowest NEFA concentrations were obtained in R721 and R07 does, respectively, and Control and R021 does showed intermediate values (*p* = 0.0158). No significant differences in NEFA levels throughout the pregnancy period were observed (*p* > 0.05).

Finally, plasma corticosterone concentration was only affected by time. The lowest concentrations of this stress indicator were observed at 21 and 28 days of pregnancy (*p* < 0.0001).

**Figure 3:**
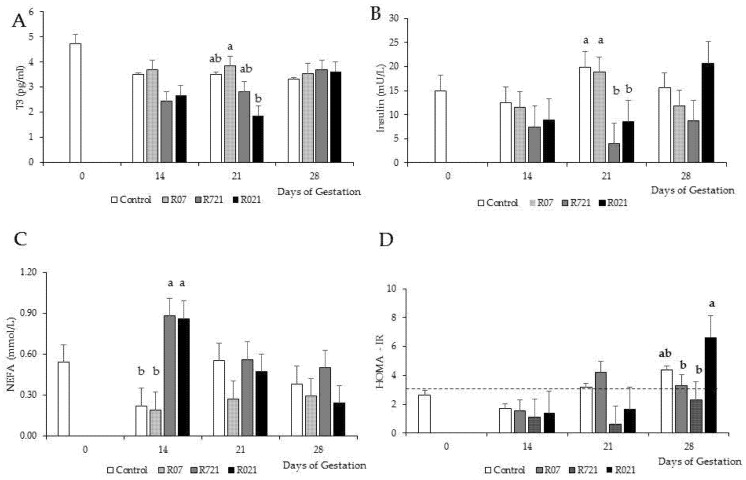
Effect of Maternal Feed Restriction and Time on (**A**) plasma triiodothyronine (T3), (**B**) insulin, (**C**) NEFA concentrations and (**D**) HOMA-IR during pregnancy of rabbits fed ad libitum (Control), restricted to 60% one week from day 0 to 7 (R07), two weeks from day 7 to 21 (R721) and three weeks from day 0 to 21 (R021) of gestation. (a–c): different letters indicate differences between experimental groups on each day of pregnancy (*p* < 0.05). Each bar on day 14, 21 and 28 shows the mean of five does, and on day 0 is the mean of 10 does. The dashed line in (D) represents the value above which HOMA-IR indicates the presence of insulin resistance.

MFR and time had significant effects on plasma concentrations of T3 (*p* = 0.0353), insulin (*p* = 0.0055), NEFA (*p* = 0.0100) and HOMA index (*p* = 0.0049) (Figure 3A–D, respectively). On day 21 of pregnancy, R021 does showed a dramatic reduction in plasma concentrations of T3 compared to R07 groups, but it was similar to Control and R721 does. The lowest plasma insulin concentrations were observed on day 21 of pregnancy in the rabbit females restricted at this time (R721 and R021). Control and R07 does had the lowest NEFA concentrations on day 14 of pregnancy. During the first half of pregnancy, HOMA-IR remained lower than 3 and by day 21 of gestation its values rose above the limit established to consider insulin resistance in Control and R07 groups. On day 28 of pregnancy, all groups showed HOMA-IR values over 3 except for group R721.

As shown in Table 5, MFR did not affect any maternal endocrine or metabolic parameters in the experimental groups during lactation (*p* > 0.05). As the lactation period progressed, T3 and glucose concentrations remained at similar values during the first two weeks of lactation, but insulin and HOMA-IR decreased on day 14 (*p* = 0.0006) compared to day 7. On weaning day, non-pregnant does had lower T3 concentrations than pregnant ones (*p* < 0.0056), but HOMA-IR, glucose and insulin concentrations were similar in both (*p* > 0.05).

Energy mobilization measured by means of plasma NEFA concentrations was similar in all groups and during all lactation. Plasma corticosterone concentrations as welfare indicators were not affected by MFR. However, significant increases in this hormone were observed as lactation progressed. No significant interactions between MFR and time in any of the studied variables were detected in this period.

## 4. Discussion

This work deals with the potential consequences of moderate MFR in different periods (1, 2 or 3 weeks) throughout gestation on reproductive outcome, including fetoplacental development and post-natal growth and survival of litters from primiparous rabbit does, and introduces further insight involving endocrine and metabolic parameters during pregnancy and the following lactation.

After different MFR periods, when fed ad libitum, all pregnant does significantly increased their feed intake. These results match with those observed in our previous studies [9,14,15]. Dams from R721 and R021 groups altogether consumed 1.3 kg and 1.8 kg less feed than the Control group during gestation, respectively. Those in the R07 group reduced their feed intake by about 300 g compared to the Control group. Considering the feed intake of Control females during the second and third weeks as the average regular consumption (227 ± 6.0 g/d and 228.3 ± 6.1 g/d), R721 and R021 does ingested 46% of the food that the Controls did. Consequently, LBW of these does was impaired, showing around a 5.2% decrease in LBW compared to Control or R07.

As expected, the number of corpora lutea assessed in the rabbits on day 28 of gestation was similar in all groups, as MFR was applied right after AI, with no impact on the ovulation rate. Similar results have been described in our previous studies where only Control and R021 treatments were compared [14,15]. Nonetheless, R07 does tended to have a higher number of fetuses, although with worse viability. In rabbits, the implantation and placentation events occur after day 6–7 post-AI [24]. In the current study, at that time R07 females experienced a significant compensatory increase in feed intake, surpassing the Control group. The slight increase in implanted fetuses observed in this group could lead to greater competition between them for uterine space and nutrients, ultimately leading to lower viability [25].

The dimensions of the fetuses were affected by the restriction, so that the longer it was, the more weight and length of the fetuses decreased, although the TD and the OND did not seem to have the same response. MFR usually causes vital organs, such as the brain or the liver, to increase their relative weight to the detriment of other organs [9,26]. This trend was not observed in this study, and other organs such as the kidneys and the liver reduced their weight in the fetuses of more restricted females. Several studies [12,27,28] assert that the longer and greater the feed deprivation is, the greater the probability of finding impaired fetal growth and development. Cappon et al. [29] performed an experiment evaluating MFR levels from slight to severe (110 g/d down to 15 g/d) and from days 7 to 19 of gestation (a period that registers from implantation to closure of the hard palate), recording the effect on fetal development. These authors showed that in severe feed deprivation, the size and weight of the fetuses were reduced, and the incidence of abortions and inadequate ossification increased. These results are in accordance with other studies [27,30] that evaluated the incidence of abortions and impairment of fetal development/growth due to feed restriction, where authors agreed that the main factors to be considered are the degree of intake restriction, its length, and more critically the period of gestation when it is applied [20], highlighting the second half of gestation as that where the energy intake of the mothers is most critical. The findings regarding fetal development comparing organ sizes of the fetuses between experimental groups at day 28 of gestation, suggest that the ration setting and periods of feed restriction applied have no long-term effects over the fetuses and later litter performance. In fact, in these experimental conditions, the brain/liver ratio was the same in all groups, and no critical variations in the relative weights of the fetal organs were observed due to the feed restriction, which supports the idea that the feed restriction applied is to be considered moderate compared to the existing records and does not induce severe impairment in fetal growth nor organogenesis.

In addition, MFR for 1, 2 or 3 weeks affected the growth of the placental structures. The largest placentas were those of R07 does, but this group was one of the least efficient, meaning that even though fetal growth directly depends on the placenta [31], in this group placentas were bigger than their fetuses. However, R721 does showed the smallest placenta because the mothers were restricted in the period when this tissue is developed. These results suggest that although placental efficiency was low in R07 and high in R721, the amount of energy provided during the period when does were fed ad libitum was enough to maintain gestation adequately, since does of these two groups were able to achieve a similar litter size at parturition, a high number of weaned kits (after adjustment), and the lowest lactation mortality rates. In this sense, Rommers et al. [28] described that a moderate decrease in energy intake during the first week of gestation, provided it is above maintenance needs, does not affect implantation rate, while in the fourth week of pregnancy, energy deprivation can generate more problems in the long-term.

Furthermore, during lactation and in the juvenile stage, our previous studies [14,15] that focused on the likely consequences on progeny of a three-week MFR (equal to the current R021 group) evidenced that the surviving offspring did not manifest any outstanding alterations in growth, serum or metabolic parameters nor feed intake during juvenile phase until puberty.

Regarding the effects of MFR on fertility and prolificacy of does, there were no adverse consequences. Although litter weight and milk production were similar between groups during lactation, a significant increase in kit mortality was observed in both the Control and R021 groups. It may be that litter adjustment performed after birth affected these results, but it is difficult to explain. Interestingly, does in group R07, despite previous lower fetal viability detected during pregnancy, had a better weaning outcome and lower kit mortality, outpacing the results of the other groups.

Concerning the mortality rate of the dams during the study (culled due to experimental needs or for other reasons such as low prolificacy, abortions, sudden deaths or mastitis), the data collected did not show significant differences between groups. They were consistent with the experimental farm’s historical data, so these casualties can be considered incidental rather than related to the experimental conditions.

As to the effects of MFR on energy metabolism, thyroid hormones were determined to evaluate metabolic adaptation to energy intake deprivation. In the current study, the mothers that underwent a longer MFR presented lower T3 plasma concentrations. During the first three weeks of gestation, a decrease in T3 and T4 was also noted, along with an increase at the end when the females were fed ad libitum. The significant treatment × time interaction observed in T3 concentrations was due to the R021 group having the lowest T3 values on day 21 of pregnancy (after three continuous weeks of moderate restriction), but by the end of gestation all groups had similar concentrations. The decrease in free T3 during periods of feed restriction usually reduces the basal metabolic rate, resulting in energy savings for the animal [32], but of all metabolism-involved hormones, T3 is the fastest to recover [33]. Thus, in the 4th week of pregnancy and during lactation, when all groups were already fed ad libitum, energy homeostasis was balanced even in the case of T3. T4 plasma concentrations during lactation rose significantly more than those observed during gestation, indicating a total recovery of the energy metabolism after MFR.

The studied relation between insulin and glucose profiles suggests that females in the first half of pregnancy, being subjected to MFR, manage the diet’s energy and body reserves. Insulin is an intermediate metabolic hormone, involved in many metabolic pathways to maintain energy homeostasis by coordinating the different axes and systems [34,35]. Furthermore, it should not be forgotten that insulin undergoes postprandial alterations and at day 14 of pregnancy the Control and R07 groups are both being fed ad libitum since day 0 and 7 of pregnancy, respectively. Afterwards, on day 21 of pregnancy, insulin increases and insulin resistance manifests with the rise in HOMA-IR in these groups, which Menchetti et al. [32] described as an adaptation to the energy demand produced by the fetuses. Following the same dynamic, decreased insulin plasma concentrations on day 21 of pregnancy in groups R721 and R021 reinforces the role of insulin in the gear of metabolism, since Buczkowska and Jarosz-Chobot [36] reported, in the same way as other authors [35], that reduced insulin plasma concentrations promotes lipolysis, which makes sense since by day 21 of pregnancy these dams had been restricted for two and three weeks, respectively, and needed to obtain energy from their body reserves. On the other hand, Fortun-Lamothe [37] described that the fetuses request high amounts of glucose at the end of gestation. Therefore, as MFR was over in R721 and R021 groups by the 4th week of gestation, these dams were obtaining glucose directly from the diet and their voluntary feed intake was considerably increased to compensate for the time of restriction, which would explain the glucose rise detected at this point, particularly in group R021, which underwent the longer MFR.

Moreover, HOMA-IR results showed significant differences between groups by the end of gestation (day 28). Particularly, R021 does displayed a critical rise in HOMA-IR values, while other groups still did not exceed the limit to be considered insulin resistant (R07 and R721). This could be due to the continuous consumption of glucose by the fetuses that forces an increase in the mother’s hepatic production of glucose by the liver during fasting to maintain plasmatic glucose sufficient to meet the needs of glucose-dependent tissues [38], which would also explain the slight but not significant rise of glucose in group R021 on day 28. Since circulating NEFA concentrations are not high enough in group R021 at this point to consider body reserve mobilization, it can be assumed that the main source of glucose was energy obtained from the diet, which at this time was being provided ad libitum for all groups.

In humans and other species, the second half of pregnancy is defined by a progressive increase in resistance to the action of insulin, so that in the third-trimester insulin sensitivity is about a third of normal and insulin levels are increased by about four times [39,40], which in our study would be equivalent to the 3rd and 4th weeks of gestation. Other authors have also obtained these same results and appreciations in rabbits [41], which concur with the data obtained in this study.

As shown by the data, MFR during gestation affected the overall energetic balance of the does and high NEFA plasma concentrations are a manifestation of body reserves mobilization. In this study, MFR affected plasma NEFA concentrations, and the highest values were observed on day 14 in the mothers with a more prolonged period of restriction at this time. This is in accordance with other studies where authors determined higher NEFA plasma concentrations in does undergoing slight to moderate MFR during similar gestation periods [32,42]. Additionally, lower circulating NEFA concentrations were observed on day 14 of gestation in Control and R07 groups, which supports the previous statement and suggests a redirection of metabolism towards a reduction of lipolysis due to higher energy intake in these groups.

Conductive to the lactation period, the main difference between groups occurred with T3 concentrations in pregnant and non-pregnant females, with lower levels in the latter. Since the third AI was performed on day 14 after parturition, increased T3 in lactating-pregnant dams may be explained by its role in tissue synthesis, particularly in the first half of pregnancy [43]. Moreover, Bober et al. [44] reported that thyroid hormones are considered necessary for cellular metabolism of the mammary gland and energy utilization, which may be an important factor in fetal development during pregnancy and milk biosynthesis, which is relevant for our study since pregnant does are overlapping lactation and 3rd pregnancy. Highlighting the slight insulin and HOMA-IR decrease that occurred on day 14 compared to day 7 post-partum, this could be explained by the stressful event of AI, which implies manipulation of the animals (removing the females from their cages, holding them by the tail, inserting the insemination catheter and injecting GnRH). When stressful situations occur, insulin levels fall, glucagon and epinephrine (adrenaline) levels rise and more glucose is released from the liver [45].

Surprisingly, no differences due to MFR between experimental groups were observed during pregnancy and lactation, something unexpected since corticosterone was assessed as a hormonal measure of stress in the animals. Some authors contend that plasma corticosterone concentrations correspond more with metabolic stress than with behavioral stress [33], which would support the assertion that in this study, MFR is only moderate and implies no metabolic depletion of the dams, since corticosterone was lower by the end of gestation. On the other hand, Menchetti et al. [32] measured cortisol levels during the pregnancies of does subjected to MFR and determined that this parameter was not altered during gestation, which can be attributed to changes in the body’s regulation during pregnancy that favor the maintenance of glucocorticoid levels, which is essential for the correct maturation of many fetal organs [46,47,48,49].

However, it is interesting to note that at the end of lactation, corticosterone levels are higher than those on day 7 of lactation. This could be explained due to the size of the kits towards the end of lactation. Kits begin to leave the nest at 14–16 days of age [50], and other authors have described that higher housing density could increase corticosterone circulating levels [51]. In these experimental conditions, by the end of lactation there are approximately 10 kits per doe weighing around 500 g, which leave the nest and occupy a lot of space in the cage, try to continue suckling and can increase the mother’s level of discomfort and explain the rise in corticosterone. Nonetheless, further studies are needed to corroborate this hypothesis.

## 5. Conclusions

The present study states that a moderate MFR (60% of the estimated voluntary feed intake) applied during 1, 2 or 3 weeks of gestation followed by ad libitum refeeding confirms previous results regarding compensatory food intake by mothers, which helps to preserve maternal energy homeostasis (thyroid hormones, glucose and insulin) and lipid metabolism (NEFA). This allowed the necessary energy supply to be delivered to the fetuses to conduct fetal growth and development satisfactorily, confirming previously obtained results as no major long-term effects in the early life development of the offspring were observed. Moreover, the compensatory feed intake granted the maintenance of an adequate productive performance in the current pregnancy and showed no effect whatsoever in the amount of milk produced, the viability of litters or the mortality rate of the dams. In addition, the corticosterone concentrations observed throughout pregnancy and lactation seem to indicate that, in our experimental conditions, the moderate MFR applied seems not to induce additional stress in dams along the gestation and lactation periods studied. Moreover, it has proven that this strategy can be profitable for the farmer as overall feed intake was lower in all experimental groups compared to the Control group. In summary, these results reveal that as long as the feed supply covers maintenance needs, moderate MFR is a suitable strategy to reduce production costs, since it implies no adverse effects on the metabolic, energetic and welfare status of the dams, and on pre- and post-natal growth of fetuses and litters, respectively.

## Figures and Tables

**Table 1:** Number and biometric parameters of fetuses of 28 days of gestational age in rabbits fed ad libitum (Control), restricted to 60% one week from day 0 to 7 (R07), two weeks from day 7 to 21 (R721) and three weeks from day 0 to 21 (R021) of gestation.

	Control n = 11	R07 n = 11	R721 n = 11	R021 n = 11	SEM	*p* Value
LBW of does (g)	4638 ^a^	4640 ^a^	4342 ^b^	4413 ^ab^	80.90	0.0254
Corpora lutea	12.2	12.3	13.1	13.4	0.58	0.4350
Total fetuses/doe	11.7	13.2	12.5	12.5	0.38	0.0814
Implantation rate ^1^ (%)	92.5	97	95.2	91	2.57	0.3684
Fetal Viability ^2^ (%)	94.6	84.9	90.3	90.1	9.13	0.0824
Fetuses weights (g)						
Total	39.7 ^a^	38.4 ^ab^	37.4 ^b^	37.0 ^b^	1.78	0.0028
Head	9.32 ^a^	9.06 ^ab^	8.75 ^b^	8.71 ^b^	0.35	0.0001
Trunk	29.0 ^a^	28.0 ^ab^	27.0 ^b^	26.8 ^b^	1.43	0.0014
Fetuses measures (mm)						
CRL	101.4 ^a^	99.5 ^ab^	98.7 ^b^	98.6 ^b^	1.83	0.0018
OND	29.0 ^a^	29.2 ^a^	28.7 ^ab^	28.3 ^b^	0.61	0.0032
BPD	19.3	19.2	19.3	19.0	0.52	0.5801
TD	20.6 ^b^	21.3 ^a^	20.8 ^b^	20.2 ^b^	0.71	0.0034
Organs weights (g)						
Brain	0.913	0.901	0.892	0.901	0.04	0.6395
Liver	2.64 ^a^	2.39 ^b^	2.36 ^b^	2.39 ^b^	0.18	0.0097
Heart	0.221	0.207	0.219	0.209	0.02	0.0734
Lungs	0.134 ^a^	0.126 ^a^	0.122 ^b^	0.126 ^a^	0.08	0.0166
Kidneys	0.359 ^a^	0.337 ^ab^	0.323 ^b^	0.307 ^b^	0.02	0.0001
Digestive tract	0.208	0.215	0.207	0.197	0.17	0.0952
Ratios						
Brain	0.024	0.024	0.024	0.025	0.00	0.4033
Liver	0.066 ^a^	0.061 ^b^	0.064 ^ab^	0.066 ^a^	0.00	0.0033
Heart	0.006 ^a^	0.005 ^b^	0.006 ^a^	0.006 ^a^	0.00	0.0028
Lungs	0.034	0.033	0.033	0.034	0.00	0.0942
Kidneys	0.009 ^a^	0.009 ^a^	0.009 ^a^	0.008 ^b^	0.00	0.0001
Digestive tract	0.053	0.056	0.056	0.053	0.00	0.1759
Brain: Liver	0.367	0.395	0.400	0.395	0.03	0.2273

LBW: live body weight. ^1^ (number of fetuses/number of corpora lutea) × 100; ^2^ (number of viable fetuses/total number of fetuses) × 100; CRL: crown–rump length; TD: thoracic diameter; BPD: biparietal diameter; OND: occipito-nasal diameter. Data are shown as least squares means. Ratio: organ weight/fetus weight. Values in the same row with different letters are significantly different. SEM: standard error of mean.

**Table 2:** Weight and dimensions of placentas from pregnant rabbits on day 28, fed ad libitum (Control), restricted to 60% one week from day 0 to 7 (R07), two weeks from day 7 to 21 (R721) and three weeks from day 0 to 21 (R021) of gestation.

	Control n = 11	R07 n = 11	R721 n = 11	R021 n = 11	SEM	*p* Value
Whole placenta (g)	5.16 ^ab^	5.21 ^a^	4.60 ^c^	4.81 ^bc^	0.30	0.0001
Labyrinth						
Weight (g)	3.27	3.51	3.30	3.33	0.80	0.0809
Length (mm)	37.40 ^a^	35.80 ^a^	35.50 ^a^	33.90 ^b^	4.28	0.0001
Width (mm)	28.50	28.40	27.70	27.40	3.72	0.1286
Thickness (mm)	5.16 ^a^	5.17 ^a^	4.70 ^b^	5.05 ^ab^	0.99	0.0053
Decidua						
Weight (g)	1.48 ^a^	1.55 ^a^	1.22 ^b^	1.40 ^a^	0.11	0.0001
Length (mm)	39.61 ^a^	37.32 ^b^	37.30 ^b^	36.09 ^b^	1.41	0.0001
Width (mm)	17.84 ^a^	18.76 ^a^	16.42 ^b^	18.39 ^a^	0.95	0.0001
Thickness (mm)	3.45 ^a^	3.52 ^a^	3.11 ^b^	3.27 ^ab^	0.26	0.0069
Placental efficiency ^1^	7.52 ^ab^	7.34 ^b^	7.86 ^a^	7.45 ^ab^	0.34	0.0162

^1^ Fetus weight/whole placenta weight. Data are shown as least squares means. Values in the same row with different letters are significantly different. SEM: standard error of mean.

**Table 3:** Productive parameters of rabbits fed ad libitum (Control), or restricted to 60% of their needs, one week from day 0 to 7 (R07), two weeks from day 7 to 21 (R721) and three weeks from day 0 to 21 (R021) in its second gestation.

	Control	R07	R721	R021		
	n = 92	n = 96	n = 92	n = 94	RMSE	*p* Value
Fertility ^1^ (%)	71.11	76.04	78.26	78.72		0.6129
Parturitions	59	69	67	68		
Litter size						
Born alive	11.01	10.98	10.05	11.02	3.17	0.2904
Stillborn	0.50	0.43	0.32	0.24	1.00	0.5514
Weaned (30 days post-partum)	10.20 ^a^	10.54 ^a^	9.59 ^b^	10.04 ^ab^	1.14	0.0004
Litter weight (g) ^2^						
At birth	605.70	619.34	580.93	643.98	142.6	0.1276
At 7 days post-partum	1306	1361	1272	1288	216.2	0.1646
At 14 days post-partum	2290	2398	2260	2276	318.1	0.1040
At 21 days p post-partum p	3314	3394	3227	3304	381.2	0.1613
At 30 days post-partum (weaning)	5782	5708	5447	5484	1129	0.3592
Milk production (kg) ^3^	8.29	8.49	8.07	8.26	9.53	0.1613
Mortality in lactation (%) ^4^	5.13 ^ab^	2.83 ^b^	3.43 ^ab^	6.32 ^a^	6.77	0.0325

^1^ Number of does pregnant after pregnancy diagnosis at 11 days post AI/total does inseminated × 100. ^2^ This parameter was obtained from 50 litters per experimental group. ^3^ Milk production = 0.75 ± 1.75 × LW21 (kg) where LW21 corresponds to litter weight at 21 days of lactation. ^4^ Mortality: [100−(kits weaned/no. of kits after litter adjustment to 8–12 kits) × 100]. Data are shown as least squares means. RMSE: root mean square error. Values in the same row with different letters are significantly different. pp: post-partum.

**Table 4:** Effect of maternal feed restriction (MFR) and time on endocrine and metabolic parameters during pregnancy of rabbits fed ad libitum (Control), restricted to 60% one week from day 0 to 7 (R07), two weeks from day 7 to 21 (R721) and three weeks from day 0 to 21 (R021) of gestation.

	Maternal Feed Restriction				Days of Pregnancy						*p* Value	
Pregnancy	Control	R07	R721	R021	0	14	21	28				
	n = 5	n = 5	n = 5	n = 5	n = 10	n = 20	n = 20	n = 20	RMSE	*p*_MFR_	*p* *_T_*_ime_	*p*_MFR × Time_
T3 (pg/mL)	3.76 ^a^	3.77 ^a^	3.19 ^ab^	3.06 ^b^	4.16 ^a^	3.07 ^b^	3.02 ^b^	3.54 ^ab^	0.79	0.0196	0.0026	0.0353
T4 (pg/mL)	4.74	4.24	5.00	5.16	5.89 ^ab^	3.66 ^b^	2.58 ^b^	7.00 ^a^	3.11	0.8525	0.0001	0.5731
T3 to T4 ratio	1.45	1.05	1.01	1.12	0.91 ^ab^	1.00 ^a^	2.06 ^a^	0.66 ^b^	1.24	0.8079	0.0022	0.5237
Insulin (mU/L)	15.73 ^a^	15.71 ^a^	9.47 ^b^	12.45 ^ab^	16.26	10.10	12.80	14.20	6.57	0.0225	0.0871	0.0055
Glucose (mg/dL)	79.94	78.00	74.49	84.13	63.82 ^b^	61.72 ^b^	76.64 ^b^	114.38 ^a^	22.34	0.6207	0.0001	0.7501
Glucose-to-insulin ratio	6.79 ^b^	7.12 ^b^	12.93 ^a^	9.17 ^ab^	4.59	9.56	10.26	11.70	6.79	0.0482	0.0802	0.2518
HOMA-IR	2.90	3.11	1.75	2.87	2.63 ^ab^	1.44 ^b^	2.40 ^b^	4.15 ^a^	1.79	0.1140	0.0001	0.0049
NEFAS (mmol/L)	0.42 ^ab^	0.33 ^b^	0.62 ^a^	0.52 ^ab^	0.54	0.54	0.46	0.35	0.27	0.0158	0.0781	0.0100
Corticosterone (ng/mL)	257.45	274.56	256.02	251.15	293.30 ^a^	363.82 ^b^	192.20 ^c^	195.85 ^c^	12.13	0.7344	0.0001	0.2519

NEFAS: no esterified fatty acids. RMSE: root mean square error. HOMA-IR: homeostasis model assessment for insulin resistance [Insulin × (glucose/18)/22.5]. Values in the same row with different letters (a, b, c) are significantly different.

**Table 5:** Effect of Maternal Feed Restriction (MFR) and Time on endocrine and metabolic parameters during lactation of rabbits fed ad libitum (Control), restricted to 60% one week from day 0 to 7 (R07), two weeks from day 7 to 21 (R721) and three weeks from day 0 to 21 (R021) of gestation.

	Maternal Feed Restriction				Days of Lactation						*p* Value	
Lactation	Control	R07	R721	R021	7	14 ^1^	30 ^2^					
	n = 5	n = 5	n = 5	n = 5	n = 20	n = 20	(P) n = 20	(NP) n = 20	RMSE	*p*_MFR_	*p*_Time_	*p*_MFR × Time_
T3 (pg/mL)	3.47	3.41	3.48	3.40	3.70 ^a^	3.56 ^a^	3.54 ^a^	2.95 ^b^	0.66	0.9830	0.0056	0.0878
T4 (pg/mL)	10.10	12.83	11.26	11.38	7.84	12.45	13.18	12.10	5.76	0.6012	0.0753	0.5611
T3 to T4 ratio	0.57	0.36	0.40	0.72	0.74	0.67	0.33	0.31	0.58	0.2853	0.0847	0.2714
Insulin (mU/L)	16.36	12.09	13.36	14.23	21.48 ^a^	12.83 ^b^	10.05 ^b^	11.68 ^b^	7.41	0.4223	0.0006	0.2539
Glucose (mg/dL)	114.33	109.93	109.31	109.51	116.51 ^a^	111.28 ^ab^	112.09 ^ab^	103.20 ^b^	12.40	0.6352	0.0199	0.2822
Glucose to Insulin ratio	10.31	21.19	11.05	17.95	6.73 ^b^	15.13 ^b^	23.34 ^a^	15.30 ^b^	15.83	0.1555	0.0469	0.0802
HOMA IR	4.73	3.32	3.70	3.95	6.26 ^a^	3.58 ^b^	2.82 ^b^	3.05 ^b^	2.17	0.3178	0.0003	0.2801
NEFAS (mmol/L)	0.26	0.28	0.25	0.29	0.24	0.26	0.27	0.30	0.12	0.8113	0.4605	0.2305
Corticosterone (ng/mL)	239.11	234.36	215.89	246.54	151.11 ^a^	217.52 ^b^	272.84 ^c^	294.44 ^c^	11.51	0.4158	0.0001	0.2334

HOMA-IR: homeostasis model assessment for insulin resistance. NEFA: no esterified fatty acids. ^1^ 3rd artificial insemination was carried out on day 14 of lactation. ^2^ Weaning day. P: pregnant. NP: non-pregnant. RMSE: root mean square error. Values in the same row with different letters are significantly different.

## Data Availability

The data presented in this study are available on request from the corresponding author.
